# Role of Echocardiography in the Management of Patients with Advanced (Stage D) Heart Failure Related to Nonischemic Cardiomyopathy

**DOI:** 10.31083/j.rcm2306214

**Published:** 2022-06-15

**Authors:** Michael Dandel

**Affiliations:** ^1^German Centre for Heart and Circulatory Research (DZHK) Partner Site Berlin, 10785 Berlin, Germany

**Keywords:** echocardiography, heart failure, heart transplantation, ventricular assist devices, myocardial recovery, weaning from assist device

## Abstract

Echocardiography (ECHO) is indispensable for evaluation of patients with terminal chronic heart failure (HF) who require transplantation or mechanical circulatory support by a left- or biventricular assist device (LVAD or BiVAD, respectively). In LVAD candidates, ECHO represents the first-line investigation necessary for a timely discovery of heart-related risk factors for potentially life-threatening post-operative adverse events, including identification of patients who necessitate a biventricular support. ECHO is also required for intra-operative guiding of VAD implantation and finding of the most appropriate setting of the device for an optimal ventricular unloading, postoperative surveillance of the VAD support, and monitoring of the RV changes in LVAD recipients. Thanks to the ECHO, which has decisively contributed to the proof that prolonged VAD support can facilitate cardiac reverse remodeling and functional improvement to levels which allow successful weaning of carefully selected patients from LVAD or BiVAD, the previous opinion that chronic non-ischemic cardiomyopathy (NICMP) is irreversible could be refuted. In patients with normalized and stable right heart catheter-derived hemodynamic parameters obtained at short-term interruptions of VAD support, ECHO has proved able to predict post-weaning long-term freedom from HF recurrence in patients with pre-implant terminal chronic NICMP. The purpose of this article is to offer an actualized theoretical and practical support for clinicians engaged in this particularly challenging and topical issue especially due to the new practical aspects which have emerged in conjunction with the growing use of long-term ventricular assist devices as bridge-to-transplantation or as destination therapy, as well as the increasing evidence that, in some patients, such VAD can become a bridge-to-recovery, allowing the removal of the device after a longer support time.

## 1. Introduction

Heart failure (HF) is a clinical syndrome of varying etiologies resulting from the loss of compensation for acute or chronic cardiac dysfunction due to structural and/or functional cardiac abnormalities, leading to low cardiac output (CO) and/or high filling pressures [1]. The management of severely symptomatic end-stage chronic HF refractory to medical therapy and implantable electronic heart rhythm management devices (i.e., stage D) is demanding, requiring specialized treatment strategies such as heart transplantation (HTx) and ventricular assist device (VAD) implantation [2]. Despite its well known limitations, HTx is still the optimal therapy for advanced refractory chronic HF. Long-term event-free survival is still better after HTx compared with mechanical circulatory support. However, the increasing use of long-term VADs as destination therapy (DT) for patients who are not eligible for HTx has substantially improved the management strategies for end-stage HF [1, 2]. In addition, there is clear evidence that some of the VADs initially designed as DT or as a bridge-to-transplantation (BTT) can turn into a bridge-to-recovery allowing their explantation after several weeks or months [3, 4, 5, 6, 7].

Echocardiography (ECHO) is a major tool for cardiac assessment in HTx and VAD candidates and is recommended as the key investigation for monitoring of VAD recipients [2]. Before implementation of a left ventricular assist device (LVAD) therapy, ECHO is indispensable for decisions regarding the need for an additional temporary or long-term right ventricular (RV) assist device, and in HTx candidates, ECHO can help in optimizing the listing of HTx (e.g., timing and prioritization) [8, 9, 10, 11].

The purpose of this article is to provide an updated overview on the usefulness and limitations of the currently available ECHO techniques for assessment of failing hearts due to primarily altered LV structure and function in patients with chronic non-ischemic cardiomyopathy (NICMP). Particular attention is paid to the importance of ECHO for detection and evaluation of ventricular reverse remodeling associated with improvement of contractile function during VAD support, as well as for weaning decision-making in patients with reversal of ventricular dilation and evidence of relevant and stable functional improvement.

## 2. Role of Echocardiography in Timely Prediction of Stage D 

Despite the beneficial effects of neurohormonal antagonists, and cardiac resynchronization therapy (CRT) with or without combined ICD, many patients eventually progress to an advanced HF stage, characterized by severe clinical symptoms, marked hemodynamic impairment, and high mortality [12, 13]. Early identification of patients at high risk for rapid cardiac deterioration leading to dependency on continuous inotrope infusions, and finally to the need for HTx or VAD implantation is crucial for successful management of end-stage HF. Continuous comprehensive and optimally timed ECHO monitoring is particularly suitable for this purpose [8, 14, 15, 16, 17, 18, 19, 20].

### 2.1 Major Echocardiographic Predictors 

Because the key features of HF resulting from cardiac structural and/or functional damages are low CO and high cavity filling pressures, it appears logical that the severity of both reduction of stroke volume (SV) and diastolic dysfunction can indicate the risk for rapid deterioration of heart function towards life-threatening stage D chronic HF. Stable patients with chronic NICMP reveal a lower base-line left atrial volume index (${\mathrm{LA}}_{\text{vol}}$-index), a longer trans-mitral E-wave deceleration time (E-DT) and lower E/A flow velocity ratios, as well as a lower early diastolic peak mitral flow to mitral annular velocity ratio (E/e’), which all indicate a less altered myocardial compliance with less restrictive LV filling [17]. The ${\mathrm{LA}}_{\text{vol}}$-index showed the highest predictive value for life-threatening cardiac worsening [17]. A large study on patients with severe HF of different etiology identified LV end-systolic volume, SV, and severe tricuspid regurgitation (TR) as independent predictors of death or the need for urgent HTx [16]. The addition of these conventional ECHO (cECHO) variables to already validated risk scores based on clinical parameters can improve de risk prediction for patients with advanced HF [16]. In a 12-month follow-up study on 100 patients with advanced chronic HF of different etiologies, where 30 patients died or necessitated urgent LVAD support, multivariate analysis identified the velocity-time integral of blood flow in the LV outflow tract (${\mathrm{VTI}}_{\text{LVOT}}$), whose reduction reflects a decrease in SV, and the systolic pulmonary artery pressure (PAPS) calculated from Doppler-derived TR velocity, as the most predictive cECHO variables [18]. Apparently paradoxical, the baseline LV ejection fraction (LVEF) in the event-free patient group was lower than in the group with adverse outcomes (27% and 32%, respectively) [17]. This finding argues against the ability of LVEF to predict sudden worsening of advanced chronic HF. In an 1-year follow-up study on 68 patients with severely reduced LVEF (18% ± 5%) due to non-ischemic dilated cardiomyopathy (DCM), where only 31 patients survived without a LVAD implantation, whereas the baseline ${\mathrm{LA}}_{\text{vol}}$-index and the RV fractional area change (${\mathrm{FAC}}_{\text{RV}}$) were associated with patient outcome, the LVEF was not significantly higher in the event-free patient group [19].

Development of pressure overload-induced RV dilation and failure (RVF) in HF syndrome triggered by LV dysfunction indicate accelerated disease progression associated with a two- to threefold increase in risk of cardiac death, regardless of the degree of LVEF impairment [21]. In patients with either reduced or preserved LVEF (HFrEF or HFpEF, respectively), the ratio between the ECHO-derived tricuspid annular plane systolic excursion and the pulmonary arterial systolic pressure (TAPSE/PASP), which allows the estimation of RV-pulmonary artery (RV-PA) coupling by plotting fiber shortening (TAPSE) vs. the force generated for overcoming the imposed load (PASP), was identified as an independent predictor of HF aggravation and fatal outcome [22]. CRT can improve RV–PA coupling in patients with mismatched TAPSE and PASP and the TAPSE/PASP ratio appeared independently associated with outcomes in CRT recipients [21]. A low TAPSE/PASP ratio (<0.45 mm/mmHg) at ≥6 months after CRT initiation in potential HTx candidates was found associated with worse survival [21]. In patients with non-ischemic cardiomyopathy, where autoantibodies against β_1_-adrenoreceptors (β_1_-AABs) are detectable in up to >80% of those with end-stage HFrEF who necessitate HTx or VAD support, immunoadsorption (IA) can improve the LVEF with >20% of the pre-IA values in 78–79% of the patients ranked as responders to IA [23]. IA appeared able to spare many of those responders to IA from HTx (or LVAD implantation) or at least delay HTx listing for years [23, 24]. These beneficial effects of IA were found particularly important in elderly people with diabetes mellitus (DM) which are less eligible for HTx and where DM is a risk factor for a worse result of VAD implantation [25]. No improvement of LVEF after IA in HTx candidates with or without DM can predict a more rapid worsening of HF [23, 24, 25].

In HF with preserved LVEF (HFpEF), where the right atrial pressure (RAP) reflects specifically the cumulative burden of abnormalities in the left heart, pulmonary vasculature, and the right heart, the RAP estimated from inferior vena cava morphology and its respiratory change [estimated right atrial pressure (eRAP)] was found particularly useful for prediction of HF worsening [15]. In a recent study, eRAP ≥8 mmHg was identified as the strongest ECHO-derived predictor of poor outcome with HFpEF followed by RV mid-diameter, E/e’ ratio, and estimated RV systolic pressure [15].

Assessments of the short-term (6 months) prognostic value of cECHO and speckle tracking echo-cardiography (STE) in HTx candidates with idiopathic DCM revealed that stable patients have at baseline higher LV end-systolic global longitudinal strain (GLS) and peak systolic strain rate values, as well as lower systolic circumferential and longitudinal intraventricular dyssynchrony indexes [8]. Stable patients reveal also longer trans-mitral E-wave deceleration times, lower E/A flow velocity ratios and LV diastolic early/late strain rate ratios, as well as higher LV late diastolic strain rate values, which all indicate a less altered myocardial compliance with less restrictive LV filling [8]. The superiority of STE over cECHO for evaluation of LV systolic dysfunction and prediction of HF aggravation was meanwhile confirmed by several other studies [19, 20, 26, 27, 28].

The reduction of GLS and increase of the ratio between the transmitral E-wave velocity and the early diastolic LV longitudinal strain rate (E/E’sr) in patients with reduced LVEF were found more predictive for cardiac worsening (mortality or urgent HTx) than the alteration of LVEF and/or the E/e’ ratio [28]. An ECHO score (including cECHO, STE and 3D-ECHO parameters) for prediction of major adverse cardiac events (including urgent need for a VAD or HTx) related to advanced chronic HF, underlines the significance of the left-sided heart filling pressure increase and the associated right-sided heart alterations (size and geometry changes plus RV systolic dysfunction) as prognostic indicators [20]. Thus, together with the ${\mathrm{LA}}_{\text{vol}}$-index, the best predictors of adverse cardiac events were: the RV sphericity index, the ${\mathrm{FAC}}_{\text{RV}}$, and the RV free-wall longitudinal strain (FWLS) [20]. A recent study revealed that particularly 3D-STE-derived LV GLS, but also 2D-STE-derived LV GLS, can be useful surrogate markers for reflecting myocardial fibrosis (MF) in patients with DCM-associated advanced HF [26]. This could explain the strong correlation of LV GLS with the RHC-derived pulmonary capillary wedge pressure (PCWP), mean pulmonary arterial pressure (mPAP), and pulmonary vascular resistance (PVR) in patients with chronic NICMP [27]. The correlation was highest for the ratio between trans-mitral E-wave velocity and GLS (E/GLS), which also correlated with the cardiac index (CI). GLS and E/GLS also proved to be good predictors of PCWP ≥15 mmHg and PVR >3 Wood units [27].

A large portion of patients with advanced HF has severe mitral regurgitation (MR), which acts as a driving force in inducing and also further aggravating the HF in a vicious cycle [14].

### 2.2 Prognostic Value of LV Size, EF and Mitral Regurgitation

Although ECHO data reflecting LV size provide important information on chronic NICMP-related LV remodeling, the cavity size cannot predict rapid deterioration of heart function toward end-stage HF. Similar degrees of LV dilation can be associated with varying degrees of systolic and diastolic dysfunction, depending mainly on the impairment of myocardial contractility, the severity of secondary MR, and the severity of filling restriction due to the reduced LV myocardial compliance. The latter is mainly a consequence of high filling pressure-induced LV overdistension and/or extensive MF, but it can also be affected by ventricular diastolic interactions [29, 30, 31]. LV dilatation is the main cause of significant MR and requires adequate medication plus close ECHO monitoring already in the early stages of LV dilation and dysfunction.

Contrary to the LV cavity size, secondary MR appeared consistently associated with higher mortality regardless of its severity and has proved to be a strong predictor of deleterious events in chronic HF initiated by a severe LV systolic dysfunction [30, 31, 32]. MR was found to be a powerful independent predictor of 12-months mortality, and the risk of death among elderly persons with moderate/severe secondary MR can be 4-fold higher than in those with absent/mild MR [30]. The optimal time for MV repair in patients with secondary chronic MR is difficult to determine because even patients with mild secondary MR have a worse prognosis and also because the smaller regurgitant volume (RegVol) due to the impaired LV systolic function, may lead to underestimation of MR severity [32, 33]. During the compensatory phase of MR, the increased preload and reduced or normal afterload as a consequence of blood regurgitation into the LA can allow a temporary preservation of the forward SV (_𝐟_SV) [34]. However, prolonged MR-related volume overload augments the LV wall stress which, by increasing the LV afterload, reduces the efficiency of systole (i.e., progressive _𝐟_SV reduction) [34, 35]. The increasing wall tension stimulates LV adverse remodeling leading thereby to further dilatation with corresponding aggravation of MR and its associated _𝐟_SV reduction with additional neurohormonal activation [14, 34, 35]. At this point, the cardiomyopathy-driven HF transits toward mitral valve (MV) driven HF, where the excessive regurgitant load can become the primary cause of death [35].

In secondary MR, in contrast to primary MR, the relationship between the LV volume overload and the EF is altered by the fact that LV dilation and dysfunction are rather the cause than the result of MR [36]. Thus, although restoration of MV competence will not be curative, timely interruption of the vicious circle of LV dilation and MR in carefully selected patients may delay the aggravation of HF [33]. According to the current guidelines, MV repair might be considered in severe symptomatic secondary MR (stage D). However, the optimal criteria for defining severe MR (Table 1, Ref. [33, 35, 36, 37, 38, 39]) and also those for the benefit/risk assessment of surgical therapy are still a controversial issue, and the identification of appropriate patients for MV repair or replacement remains challenging [33, 36, 37, 38, 39, 40]. The fact that secondary MR is dynamic and load dependent additionally complicates the evaluation of its severity [32, 35, 38]. Grading of functional MR is also more challenging due to the lack of structural abnormalities of the MV leaflets and chords and also because symptoms, LV and LA dilation, pulmonary congestion, as well as alterations of the pulmonary venous flow pattern, may be caused by the underlying cardiomyopathy, and are thereby less useful for establishing the severity of MR [35].

**Table 1.** **Echocardiographic diagnostic criteria for severe mitral regurgitation**.

Guidelines and studies	Threshold values of major ECHO-derived variables which indicate severe MR		
	EROA	RegVol	RegFrac
Nishimura *et al*. [33]	≥40 mm²	≥60 mL/beat	≥50%
2017 AHA/ACC guidelines			
Baumgartner *et al*. [37]	≥20 mm²	≥30 mL/beat	
2017 ESC/EACTS guidelines			
Zoghbi *et al*. [38]	≥40 ${\mathrm{mm}}^{2}$	≥60 mL/beat	≥50%
2017 ASE guidelines	30–39 ${\mathrm{mm}}^{2}$ if 3 of 4 other specific ECHO criteria* are present or if elliptical ROA	45–50 mL if 3 of 4 other specific ECHO criteria* are present or if elliptical ROA	40–49% if 3 of 4 other specific ECHO criteria* are present or if elliptical ROA
Bartko *et al*. [36] study	≥30 ${\mathrm{mm}}^{2}$	≥45 mL/beat	≥50%
2019 Unifying concept			
Bonow *et al*. [35]	≥40 ${\mathrm{mm}}^{2}$	≥60 mL/beat	≥50%
2020 ACC Expert Consensus	30–39 ${\mathrm{mm}}^{2}$ if 3 of 4 other specific ECHO criteria* are present or if elliptical ROA	45–50 mL if 3 of 4 other specific ECHO criteria* are present or if elliptical ROA	40–49% if 3 of 4 other specific ECHO criteria* are present or if elliptical ROA
Vahanian A. *et al*. [39]	≥40 ${\mathrm{mm}}^{2}$	≥60 mL/beat	≥50%
2021 ESC/EACTS guidelines	or ≥30 ${\mathrm{mm}}^{2}$ if elliptical ROA	or ≥45 mL if low flow conditions	

ECHO, echocardiography; MR, mitral regurgitation; EROA, effective regurgitant orifice area; RegVol, regurgitant volume; RegFrac, regurgitant fraction; ROA, regurgitant orifice area; AHA, American Heart Association; ACC, American College of Cardiology; ASE, American Society of Echocardiography; ESC, European Society of Cardiology; EACTS, European Association for Cardio-Thoracic Surgery. * the 4 criteria are: vena contracta ≥7 cm or ≥5 ${\mathrm{cm}}^{2}$, proximal isovelocity surface area ≥1 cm at Nyquist 30–40 cm/s, central large regurgitant jet ≥50% of left atrial area and, pulmonary vein systolic flow reversal.

Because no single ECHO parameter is sufficient for quantifying MR in individual patients, integration of multiple parameters is mandatory for assessment of MR severity [35, 36, 40]. However, because the choice of the most appropriate therapeutic strategy for severe HF related to chronic NICMP associated with severe secondary MR (e.g., MV repair, VAD implantation or listing for HTx) is based on multiple factors; ECHO-derived data, although indispensable, represent only a part of the information requested for a final decision-making.

The impact of MR on the validity of EF for the evaluation of LV contractile function was already recognized decades ago [41]. In view of the high incidence of secondary MR in NICMP, this problem needs particular consideration in order to avoid misinterpretation of measurements, because LVEF can be misleading in patients with severe secondary MR [39]. The reason for the negative impact of MR on the validity of EF as a parameter of LV contractile function is the fact that, in the presence of MR, the _𝐟_SV is not anymore the difference between the end-diastolic and end-systolic volume (EDV-ESV) because EDV-ESV will become the sum of two volumes (i.e., _𝐟_SV + RegVol) and, with aggravation of MR, the regurgitant fraction (RegFrac) increases to the detriment of _𝐟_SV [41, 42, 43, 44, 45, 46]. By inducing LV pre-load and afterload changes, MR increases the EF values leading thereby to overestimation of LV pump function which carries the risk that potential candidates for MV repair could be referred too late for intervention [34, 43]. Two large studies revealed no differences in the LVEF between patients with moderate and those with severe secondary MR (i.e., 25% in both groups, in both studies), although those with severe MR had a significantly higher risk of mortality [31, 36]. MR-induced LVEF increase can also be the explanation for the frequently observed reductions of LVEF after MV repair or replacement despite the increase of the _𝐟_SV [41, 42, 43]. In the presence of MR it is necessary to measure the _𝐟_SV (i.e., VTI of the systolic jet measured with the pulsed-wave [PW] Doppler in the LVOT multiplied with the LVOT area), because in these patients, _𝐟_SV and EF provide together more relevant information on LV pump function [45]. In the ESC/EACTS 2017 guidelines, LVEF between 15% and 30% was considered as one of the criteria for selection of patients who may be considered for MV repair [37]. This would mean that an adult with a LVEDV of 300 mL, a LVEF of 20% and a RegFrac of 50% might be a candidate for MV repair, even though his low _𝐟_SV and forward LVEF (i.e., 30 mL and 10%, respectively), indicate the necessity of intensive care and, if feasible, a MCS but certainly not MV repair. The 2021 ESC/EACTS guidelines emphasize the misleading impact of MR on LVEF, and LVEF values between 15% and 30% are not anymore recommended as a selection criterion for MV repair in secondary severe MR resulted from cardiomyopathy-induced LV dilation [39].

Overrating of LV pump function can be prevented by quantification of the forward EF (_𝐟_EF) using the formula _𝐟_EF(%) = [_𝐟_SV/EDV] ×100 which can increase the predictive value of EF for patient outcome with LV systolic dysfunction of different etiologies and improve the management of HF patients [43, 44, 47]. An important prerequisite for the use of this strategy is an accurate measurement of the LV-EDV which may require 3D-ECHO. ${\mathrm{SV}}_{\text{f}}$ quantification also allows the calculation of the RegVol, which cannot be reliably estimated directly due to the usually high turbulence and eccentricity of the regurgitation jets [42, 43, 44]. By using the formula: RegVol = EDV – (ESV + _f_SV), the RegVol can be easily calculated [43]. For this calculation, however, it appears advisable to measure the EDV and ESV by 3D-ECHO which allows more precise computation of the LV volumes [48, 49].

MR also affects the validity of E/e’ by increasing the velocity of early diastolic displacement of the mitral annulus in accordance with the increase of diastolic flow volume across the mitral valve [50]. This can lead to underestimation of LV diastolic dysfunction and can adversely affect the value of E/e’ for timely prediction of rapid deterioration of heart function toward end-stage HF.

In patients with advanced HFpRF it is likely that the frequently detectable mild or moderate MR, which appeared associated with greater hemodynamic severity of cardiac dysfunction, is caused by progressive mitral annulus stretching as a result of LA remodeling and dysfunction (i.e., LA myopathy) [51]. Because even mild functional MR is associated with upstream impairments in pulmonary vascular function, RV dysfunction and higher incidence of TR, its presence in HFpEF (even without atrial fibrillation) can be considered as a clear evidence of a higher risk for further cardiac worsening [51].

## 3. Role of Echocardiography in Referral for Transplantation

Timely referral of suitable patients is a key to good outcomes of HTx. The persistence of a low CO syndrome unmanageable by the available therapeutic options (e.g., maximal medical therapy, CRT, MV repair), is a main indication for HTx and, therefore, close monitoring of SV by ECHO can be particularly useful for timely placement of patients on the HTx waiting list [14]. First it has to be decided whether the patient is eligible for both HTx and VAD implantation, or only for one of them. Those eligible for HTx should be timely listed, but VAD support (LVAD ± additional RV support) as BTT should be considered in unstable patients [14]. Because some of the patients, eligible for LVAD implantation but not for HTx, may become HTx candidates in the future (e.g., those with reversible secondary pulmonary hypertension), their identification for reconsideration of HTx after LVAD implantation is essential for a best possible therapy [14, 52, 53]. All this presupposes complex multi-disciplinary investigations and integrative interpretation of a large variety of clinical, hemodynamic, imaging and laboratory data, which alone would be unable to predict patient outcome. Reversibility of pre-HTx pulmonary hypertension (mPAP >25 mmHg, PVR >2.5 Wu, and trans-pulmonary pressure gradient [ΔP] >12 mmHg despite optimal medical therapy) is not predictable by ECHO [53].

## 4. Role of Echocardiography in Ventricular Assist Device Therapy 

There is general agreement about the importance of ECHO in ascertaining the necessity and feasibility of LVAD support, taking decisions about the need for an additional assist device also for the right-sided heart in LVAD candidates, intraoperative guiding of VAD implantation and adjustment of the assist device flow, as well as post-operative monitoring of the ventricular support including accurate monitoring of the right-sided heart in LVAD recipients (Table 2, Ref. [3, 4, 5, 6, 7, 11, 22, 54, 55, 56, 57, 58, 59, 60, 61, 62, 63, 64, 65, 66, 67, 68, 69, 70, 71, 72, 73, 74, 75, 76, 77, 78, 79, 80, 81, 82, 83, 84, 85, 86, 87, 88, 89, 90, 91, 92, 93, 94, 95, 96, 97, 98, 99, 100, 101, 102, 103, 104, 105, 106, 107, 108, 109, 110, 111, 112, 113, 114, 115, 116, 117, 118, 119, 120]) [2, 54, 121, 122, 123]. In addition, ECHO is the first-line tool for detection and estimation of ventricular reverse remodeling and functional improvement in response to the VAD support, selection of potential weaning candidates, weaning decision-making and monitoring of cardiac function after VAD explantation [54].

**Table 2.** **Major benefits and limitations of echocardiography for optimization of LVAD therapy in patients with end-stage chronic non-ischemic cardiomyopathy**.

Role of ECHO	Usefulness	Challenges and limitations
Selection of LVAD candidates [55, 56, 57, 58, 59, 60, 61]	- Detection of cardiac abnormalities (e.g., thrombi, PFO, endocarditis, valvular abnormalities) and aortic diseases (e.g., aneurisma, atheroma, coarctation) that increase the risk for complications.	- The low AR velocities resulting from the low ∆P between the aorta and the LV during the diastole hamper the quantification of AR. Routine calculation of both _f_SV and regurgitant fraction in all LVAD candidates can avoid underestimation of the AR.
Pre-operative prediction of RVF after LVAD implantation [11, 22, 60, 62, 63, 64, 65, 66, 67, 68, 69, 70, 71, 72, 73, 81, 85, 88]	- Pre-operative RVEDD >55 mm, RV ${\mathrm{S/L}}_{\text{ED}}$ >0.57, RV/LV diameter-ratio ≥0.75, LA volume index >38 mL/m², TAPS’ <8 cm•$s^{-1}$, TAPSE <7.5 mm, ${\mathrm{FAC}}_{\text{RV}}$ <31%, PSSL <–9.6%, PSSrL <0.6•$s^{-1}$, TR grade >2, and Δ$P_{\text{RV-RA}}$ <35 mmHg, were identified as risk-factors for RVF.	- RVEDD, ${\mathrm{FAC}}_{\text{R}}$, TAPSE and TR >grade 2 were not identified in all studies as significant risk-factors for RVF. The other ECHO-derived parameters can predict freedom from RVF with relative high probability (NPV between 76% and 96%). Prediction of RVF appears less reliable. Only the global PSSrL (PPV 95%) and Δ$P_{\text{RV-RA}}$ (PPV 76%) were also found useful for prediction of RVF.
	- The use of integrative parameter combinations, which allow the evaluation of RV alterations in connection with RV afterload, appeared more reliable for prediction of RV anatomical and functional responses to the LVAD support. TAPSE/PAPsyst, PSSrL • Δ$P_{\text{RV-RA}}$ and the ${\mathrm{LAI}}_{\text{RV}}$ can substantially improve the contribution of ECHO to the preoperative prediction of the persistence or new occurrence of RVF after LVAD implantation. The ${\mathrm{LAI}}_{\text{RV}}$ appeared also useful for detection of patients with such an impaired RV adaptability to load that will be insufficient to prevent RVF, even at normal PVR.	- The distinctly load dependency of RV geometry, size and function is the main reason for the low predictive value of the individual RV anatomical and/or functional ECHO-derived parameters for the persistence or new occurrence RVF during LVAD support. This also includes the impact of TR, which can induce RVEF, ${\mathrm{FAC}}_{\text{RV}}$ and TAPSE changes with a misleading impact on the assessment of RV contractile function. Prediction of RV responses to LVAD support only by ECHO is mainly limited by the fact that the responses depend not only on the reversibility of RV myocardial alterations, but also on the reversibility of pathologic circulatory and metabolic changes related to the long-term persistence of congestive HF and the consequent end-organ failure (especially kidney and liver). ECHO allows no reliable distinction between afterload-induced and myocardial contractile dysfunction-induced RVF.
Decision on the necessity for additional RVAD [11, 22, 54, 65, 69, 73, 74, 75, 76, 77, 78, 79, 80, 81, 82, 83, 84, 85, 86, 87, 88]	- ECHO-derived data on cardiac anatomy and function are mandatory for decision making in favor or against the additional consideration of a mechanical support also for the RV. ECHO mainly enables the identification of patients without the need of RVAD support after LVAD implantation. The ${\mathrm{LAI}}_{\text{RV}}$, which enables the identification of patients with massively impaired RV adaptability to load (unable prevent RVF, even at normal PVR), can be particularly useful for detection also of those who need biventricular mechanical support.	- The prediction of the need for RV mechanical support by ECHO and/or by any other available tool for evaluation of the right-sided heart is particularly challenging because many patients with post-LVAD RVF can improve and finally even normalize their RV function thanks to the elimination of pulmonary congestion and the possibility to use maximum pulmonary vasodilation therapy. Another important aspect is the fact that patients who need post-operatively an emergent implantation of a mechanical support for the RV are only a minority of LVAD recipients with RVF and the pre-operative identification of these patients still remains a major challenge because both unnecessary BiVAD implantation and delayed transition from LVAD to BiVAD support may have adverse impact on patient outcome.
Assessment of recovery and weaning decision-making [3, 4, 5, 6, 7, 54, 57, 81, 89, 90, 91, 92, 93, 94, 95, 96, 97, 98, 99, 100, 101, 102, 103, 104, 105, 106, 107, 108, 109, 110, 111, 112, 113, 114, 115, 116, 117, 118, 119, 120]	- ECHO is the main tool for selection of weaning candidates, evaluation of the functional relevancy and stability of recovery, and together with RHC, also mandatory for decision-making in favor of or against VAD explantation.	- No single anatomical or functional ECHO parameter allows alone the evaluation of recovery.
		- Multiparametric ECHO evaluation of cardiac anatomy and function plus integrative interpretation of measurements are essential requirements for assessment of recovery. Pre-explant ECHO can predict post-explant outcome only in patients with normal off-pump RHC data.

ECHO, echocardiography; LVAD, left ventricular assist device; PFO, patent foramen ovale; ΔP, pressure gradient; AR, aortic regurgitation; _𝐟_SV, forward stroke volume; RVF, right ventricular failure; RV, right ventricle; RVEDD, RV end-diastolic diameter; ${\mathrm{S/L}}_{\text{ED}}$, end-diastolic short/long axis ratio; TAPSE and TAPS’, tricuspid lateral annulus peak systolic amplitude and Doppler-derived peak velocity; respectively; ${\mathrm{FAC}}_{\text{RV}}$, RV fractional area change; TR, tricuspid regurgitation; PSSL and PSSrL, global peak systolic longitudinal strain and strain rate; respectively; Δ$P_{\text{RV-RA}}$, pressure gradient between RV and RA; NPV and PPV, negative and positive predictive value; respectively; ${\mathrm{LAI}}_{\text{RV}}$, RV load adaptation index; BiVAD, biventricular assist device; RHC, right heart catheterization.

### 4.1 Candidate Selection for LVAD Implantation 

Preoperative ECHO is indispensable for LVAD candidate selection which is a demanding task due to the fact that end-stage HF caused by severe LV dysfunction is a multifaceted syndrome with multiple risk factors for an unfavorable course of LVAD therapy [55, 56, 57, 58]. Small LV chamber dimensions in patients with restrictive cardiomyopathy pose particular challenges for a LVAD implantation. Thus, LVEDD <4.5 cm was identified as a risk factor for LVAD suction events and thrombosis [14]. This aspect is essential for optimal therapeutic decision-making in severe HFpEF, particularly in patients with hypertrophic cardiomyopathy [14]. The more recently introduced micropump-based circulatory support systems, where the pump inflow derived from the LA allows active decompression of the LA and the pulmonary circulation with simultaneous improvement in systemic blood flow, can be a safer option for BTT in such patients [124].

ECHO is the preferred screening tool for detection of pathological alterations of the heart and thoracic aorta that enhance the risk for potentially life-threatening events, or may even contraindicate LVAD insertion [58]. However, in patients with evidence of aortic abnormalities, ECHO alone can be insufficient for accurate assessment which will necessitate the use of additional imaging techniques (e.g., CT scan).

TTE and TEE (transthoracic and transesophageal echocardiography, respectively) are particularly useful for pre-implant detection of possible thrombi, patent foramen ovale (PFO), valve abnormalities, endocarditis or aortic diseases. Special attention should also be paid on indications that suggest the presence of PFO [56, 57, 58, 59].

Exclusion of relevant (≥moderate degree) aortic regurgitation (AR) is mandatory. Because AR impairs LVAD support and must therefore be eliminated concurrently with the LVAD insertion [59, 60, 61]. AR severity grading before LVAD implantation is difficult because of the low SV (due to the reduced LV systolic pressure) and the reduced velocity of the regurgitant flow due to the low diastolic ΔP between the aorta and the LV (as a result of the reduced diastolic pressure in the aorta and the increased LV diastolic pressure). Consequently, the regurgitant volume can also be relatively small, despite a high RegFrac, which often leads to underestimation of AR severity. Therefore, it is advisable to quantify both RegFrac and _𝐟_SV in all patients referred for LVAD implantation [58]. The search for AR is crucial in the presence of aortic root dilatation, aortic valve (AV) structural alterations, or eccentric regurgitation [55]. Because more than moderate TR is a relevant risk factor for right-sided heart failure (RHF) after LVAD insertion, tricuspid valve repair (TVr) should be considered at the time of LVAD insertion [58, 60]. In patients with TR 3+, concurrent LVAD implantation and TVr showed similar short-term outcome results with the implantation of a biventricular assist device (BiVAD) [60]. Failure of TVr in patients with concurrent LVAD implantation and TVr appeared independently associated with late RHF, and RHF-free survival probability was found higher in patients without TVr failure [61]. Pulmonary regurgitation (PR) of moderate degree can be well tolerated by patients with optimal LVAD support. On the contrary, in LVAD candidates who necessitate additional mechanical support also for the RV, already a moderate PR necessitates valve repair during VAD surgery [56, 58]. Because certain aortic abnormalities (aneurysm, atheroma, coarctation) can complicate or even contraindicate LVAD insertion, their preoperative detection can be crucial for a successful LVAD therapy [56, 58].

### 4.2 Prediction of Successful LVAD Therapy

RVF is a major cause of morbidity and mortality in LVAD recipients [54]. Because of their particularly strong load dependency, the RV size, geometry, and function are often altered already before LVAD implantation. Reverse remodeling and functional improvement of the RV during mechanical support of the LV depend on the reversibility of both the RV myocardial alterations and the pathologic circulatory and metabolic changes triggered by imbalanced neurohumoral/inflammatory reactions to the insufficient CO and the HF-related end-organ failure (especially renal and liver failure) [54, 125]. Conversely, the reversibility of pathologic neurohumoral activation and end-organ failure during mechanical LV support depend on the reversibility of RV dysfunction. Prediction of the impact of mechanical LV support on RV function and on patient outcome without additional RV support becomes therefore particularly difficult. This also explains the fact that the majority of the scoring-systems currently used for supporting the decision in favour of an LVAD or BiVAD therapy are based rather on parameters related to the pre-implant severity of HF-related multiorgan dysfunction than on parameters directly related to RV contractile performance and its potential reversibility [10, 54, 62, 63, 126, 127]. This could be the reason for the differences regarding the risk prediction for RVF during LVAD support revealed by different studies using the same scoring-system which did not include parameters of right-sided heart anatomy and function.

Several risk-scores (with and without incorporated ECHO-parameters) for preoperative prediction of RVF during LVAD support can also predict the mortality risk after LVAD insertion [63, 64, 65, 127, 128, 129, 130, 131]. The best capability to identify LVAD candidates at high risk for postoperative death was found for complex scoring-systems that include preoperative hemodynamic, laboratory and clinical parameters that reflect mainly end-organ dysfunction which on its part can remain a serious risk for death even after improvement of RV function due to the LVAD-promoted reduction of the PVR [57, 63, 129, 130, 131]. Nevertheless, addition of ECHO-derived variables for evaluation of RV size, geometry and function to different risk-scores based exclusively on symptoms parameters and reflecting the preoperative severity of HF-related multiorgan dysfunction, can improve the preoperative prediction of patient outcome after LVAD implantation [64, 66, 67].

Preoperative prediction of myocardial recovery during prolonged LVAD or BiVAD support, sufficient for later explantation of the VAD is not possible, neither by ECHO, nor by any other investigation [4, 5, 6, 17, 54].

### 4.3 Prediction of the RV Recovery Potential in LVAD Candidates 

ECHO allows pre-implant identification of LVAD candidates with and without the necessary preconditions to become and/or remain free from RVF after LVAD implantation, because those with inadequate responses of the RV to the support of the LV have already pre-operatively more altered morphological and functional right-sided heart parameter values [62, 63, 64, 67, 68, 126, 132]. Thus, patients with RVF during mechanical LV support showed preoperatively larger RV end-diastolic diameters (RVEDD) with greater S/L (short/long axis) ratios, greater RV/LV diameter ratios, lower RVEF and ${\mathrm{FAC}}_{\text{RV}}$, lower TAPSE and tricuspid lateral annulus peak velocity of systolic displacement (TAPS’), lower peak systolic longitudinal strain and strain rate (PSSL and PSSrL, respectively) values at the RV free wall, more severe TR and higher systolic ΔP between the RV and RA (Δ$P_{\text{RV-RA}}$) [11, 60, 62, 63, 64, 65, 66, 67, 68, 69, 70, 126, 127, 132, 133, 134, 135, 136]. Although all these preoperative ECHO parameter alterations were rated as risk-factors for the presence of RVF after LVAD insertion, not all of them were recognized in all studies as relevant risk-factors for RVF [49, 66, 71, 72, 137, 138]. This could be explicable by a possible different impact of certain well known intrinsic limitations of ECHO for RV assessment, but also by differences between different studies regarding the applied selection criteria for LVAD implantation and/or in defining RVF [49, 139].

### 4.4 Decision Regarding the Need for Additional RV Support

Preoperative identification of LVAD candidates who necessitate mechanical assistance for both ventricles is essential for optimal postoperative outcomes. Compared to BiVADs, LVADs are safer for the patients and offer a higher quality of life, but even if LV myocardial alterations and dysfunction were the primary cause of advanced HF, RV dysfunction of different severity is nearly always present. Unfortunately, although several ECHO parameter alterations which are recognized as risk-factors for RHF after LVAD implantation can predict preoperatively the absence of RVF after LVAD implantation, only few of them can also predict the occurrence of RVF during the LVAD support [11]. Prediction of the need for additional mechanical support also for the RV is difficult, because many patients with post-LVAD RVF can improve and finally even normalize their RV function thanks to the elimination of pulmonary congestion and the possibility to use a maximum pulmonary vasodilation therapy [11, 69, 73]. However, such prediction would be important because LVAD recipients who receive a RVAD much later after LVAD surgery have a less favorable outcome than those with simultaneous LVAD and RVAD insertion [54, 139]. It should also be considered that in many patients at high risk for RHF after LVAD surgery, the concurrent insertion of a durable LVAD and an easily removable temporary RVAD can avoid the implantation of a durable BiVAD [54, 139].

There are major difficulties and methodological weaknesses in the evaluation of the RV by ECHO which can greatly influence the diagnostic and prognostic reliability of this technique in VAD candidates [74, 75, 76, 77, 78]. Because of the distinctly high dependency of RV size, geometry and pump function on hemodynamic loading conditions, a major limitation for the diagnostic and prognostic value of individual RV parameters is their frequent alteration already before any impairment of RV myocardial contractility. This contributes to the limited predictive value of pre-operatively measured ECHO parameters which had proved to be relevant risk-factors for RV failure after LVAD insertion. Parameters like ${\mathrm{FAC}}_{\text{RV}}$, RVEF, and TAPSE, as well as RV systolic wall motion and myocardial deformation velocity, can significantly decrease with increasing PVR even without alteration of myocardial contractility. In addition, relevant TR can cause misleading ${\mathrm{FAC}}_{\text{RV}}$, TAPSE, and RVEF changes, which can impede the assessment of RV contractile function [54, 79, 80]. Therefore, without the interpretation of the above parameters in relation to the PAP, Δ$P_{\text{RV-RA}}$, or PVR, ECHO assessment of RV function may become doubtful and this could also explain its rather modest preoperative predictive value for the presence of severe RVF after LVAD insertion [11, 79].

The ECHO-based assessment of the RV can be improved by using also different combinations of parameters which include measures that reflect the RV afterload [11, 22, 80, 81, 82, 83, 84, 85, 139, 140]. Because the RV stroke work index (${\mathrm{SWI}}_{\text{RV}}$) calculated from RHC measurements appeared superior to different individual ECHO-derived parameters used for assessment of the RV in patients with end-stage congestive HF, it was suggested that the ECHO-derived ${\mathrm{SWI}}_{\text{RV}}$ might be a potentially useful combined parameter for RV assessment in relation to its afterload [83, 140]. However, because ECHO estimation of mPAP and mean RA pressure is difficult, the ${\mathrm{SWI}}_{\text{RV}}$ calculated from measurements obtained by ECHO is rarely used in the clinical praxis. In the meantime however, two easy calculable ECHO-derived composite parameters were found suited to be used as surrogates for the RHC-derived ${\mathrm{SWI}}_{\text{RV}}$ [83, 140]. One of them is the “simplified RV contraction-pressure index” (sRVCPI), which incorporates TAPSE and load (i.e., sRVCPI = TAPSE • Δ$P_{\text{RV-RA}}$), the other is the “RV stroke work” (RVSW) which incorporates the SV and load (i.e., RVSW = 4•[peak TR jet velocity]^2^•SV) [84, 140]. Both correlate closely with the RHC-derived ${\mathrm{SWI}}_{\text{RV}}$ and the sRVCPI also revealed a high sensitivity and specificity to predict a ${\mathrm{SWI}}_{\text{RV}}$ reduction [78, 79]. The usefulness of sRVCPI and RVSW for RV assessment in LVAD candidates remains to be assessed.

Other composite ECHO-derived parameters and indexes, which combine longitudinal displacement and afterload (i.e TAPSE/PAPS and TAPSE/PVR), or myocardial shortening velocity and load (i.e., “afterload corrected PSSrL”) were also found appropriate for evaluation of the RV myocardial contractile ability [11, 77, 84, 140]. The ratio of TAPSE/systolic PAP appeared to be an independent predictor of mortality in patients with congestive HF due to primary impaired LV function [65]. Because PVR has a decisive impact on RV systolic function, its inclusion into ECHO-derived composite parameters can be useful for evaluation of the relationship between increased afterload and RV function [22]. A composite ECHO-derived parameter which reflects that relationship in a simplified manner is the “RV ejection efficiency” (RVEe), defined as RVEe=TAPSE/PVR [22]. Using TAPSE as a surrogate for RV ejection and ECHO-derived PVR (PVR=TR peak velocity/RV outflow tract VTI) as a surrogate for the RHC-derived PVR, the easy calculable RVEe could be useful for assessment of RV functional abilities [86, 87]. However, future studies are needed to determine whether the ECHO-derived RVEe can be able to predict RV function post-LVAD RV function.

The RV free wall “afterload-corrected PSSrL”, defined as PSSrL • Δ$P_{\text{RV-RA}}$, allows the detection of an afterload mismatch already before the reduction of RV contractility [11, 80, 88, 139]. Thus, due to the load dependency of the myocardial shortening, an increase in RV afterload leads to the decrease of the RV free wall PSSrL. However, as long as the RV contractility remains unaltered, due to the simultaneous Δ$P_{\text{RV-RA}}$ increase in response to the high afterload, the afterload-corrected PSSrL remains relatively stable. As soon as the afterload increase overwhelms RV ability to adapt its contractile function correspondingly (afterload mismatch), there will be also a reduction in Δ$P_{\text{RV-RA}}$ (by increase of RA pressure), even before finally RV systolic pressure will also drop due to progressive impairment of RV contractility, which leads to a more pronounced reduction of Δ$P_{\text{RV-RA}}$ and thereby to massive reduction of the afterload-corrected PSSrL.

Evaluation RV ability to improve or even normalize its function after lowering its afterload is enabled by calculation of the ${\mathrm{LAI}}_{\text{RV}}$ which is based on the relationship between the Δ$P_{\text{RV-RA}}$, which reflects the RV loading conditions, and the RV end-diastolic volume per long-axis length (${\mathrm{EDV/L}}_{\text{ED}}$), which reflects the degree of cavity remodeling:

(1) $${\mathrm{𝐋𝐀𝐈}}_{\mathrm{RV}}=\frac{\Delta \,{𝐏}_{\mathrm{RV}-\mathrm{RA}}}{\mathrm{𝐄𝐃𝐕}/{𝐋}_{\mathrm{ED}}}\approx \frac{{\mathrm{𝐕𝐓𝐈}}_{\mathrm{TR}}}{{𝐀}_{\mathrm{ED}}/{𝐋}_{\mathrm{ED}}}=\frac{{\mathrm{𝐕𝐓𝐈}}_{\mathrm{TR}}\,\left(\mathrm{cm}\right)\cdot {𝐋}_{\mathrm{ED}}\,\left(\mathrm{cm}\right)}{{𝐀}_{\mathrm{ED}}\,\left({\mathrm{cm}}^{2}\right)}$$

**Fig. 1.**
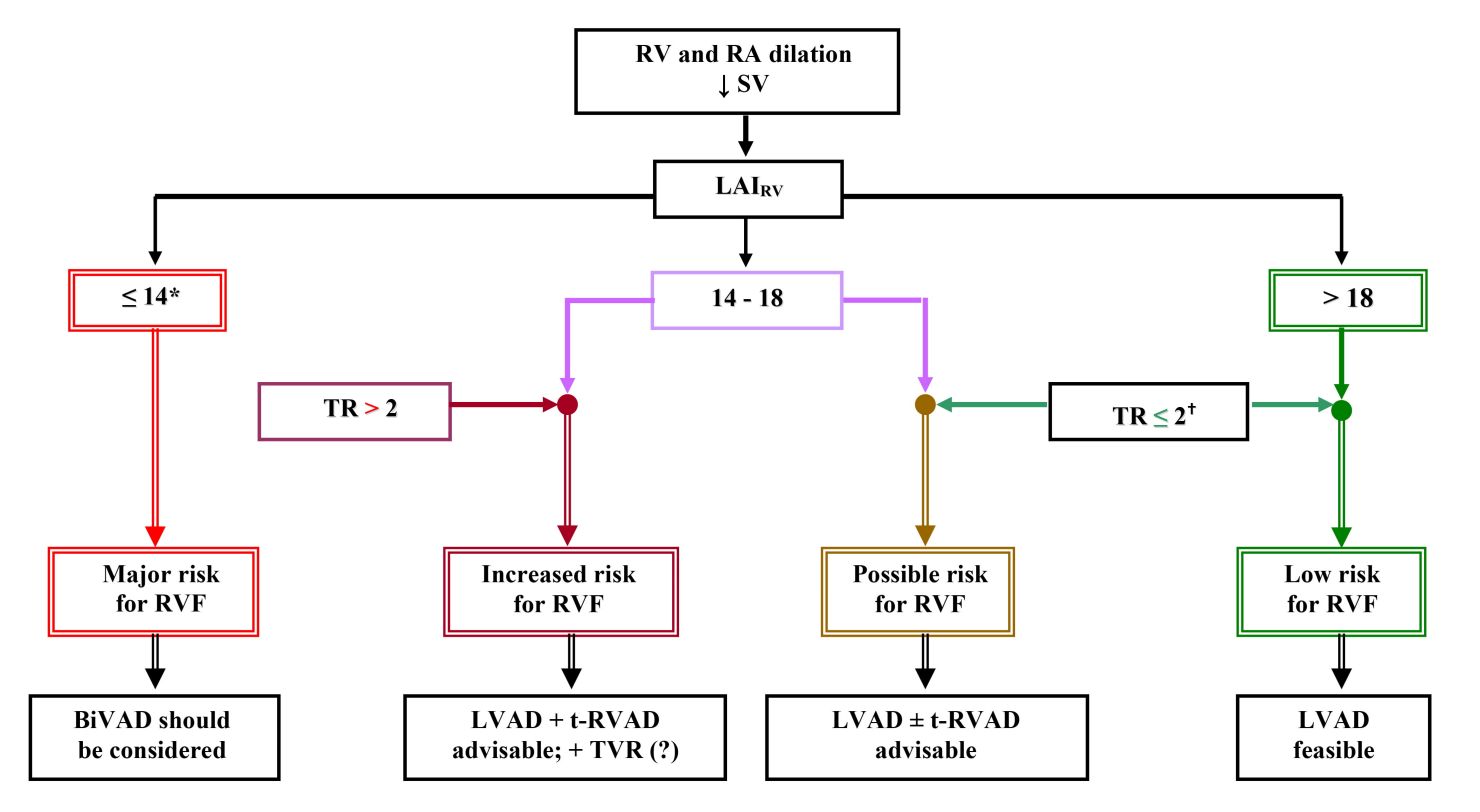
**ECHO-based clinical algorithm for device selection in patients with end-stage non-ischemic chronic cardiomyopathy [9, 11, 79, 88, 91]**. ECHO, echocardiography; RV, right ventricle; RA, right atrium; ↓SV, reduced stroke volume; LAI, load adaptation index; Δ$P_{\text{RV-RA}}$, pressure gradient between RV and right atrium; TR, tricuspid regurgitation; RVF, RV failure; BiVAD, biventricular assist device; LVAD, LV assist device; t-RVAD, temporary RV assist device; TVR, tricuspid valve repair. * ${\mathrm{LAI}}_{\text{RV}}$ ≤14 indicates severely impaired adaptability to afterload (insufficient RV systolic pressure increase at simultaneously increasing RA pressure and disproportionate adverse impact on RV geometry); ^†^ no relevant TR indicates less elevated RA pressure, as well as less dilated tricuspid valve ring, and thus also higher chances of a RV reverse remodeling and functional recovery during LVAD support.

The replacement of Δ$P_{\text{RV-RA}}$ by the TR velocity-time integral (${\mathrm{VTI}}_{\text{TR}}$) and the RV end-diastolic volume (EDV) by the RV end diastolic area ($A_{\text{ED}}$) enabled the obtainment of a dimensionless index of comparable diagnostic validity [11, 80, 139]. The use of ${\mathrm{VTI}}_{\text{TR}}$ instead of Δ$P_{\text{RV-RA}}$ is possible without restrictions because Δ$P_{\text{RV-RA}}$ is calculated from the mean velocity of the TR-jet, and ${\mathrm{VTI}}_{\text{TR}}$ has the advantage to include also the duration systolic loading. The strong correlation between the ECHO-derived ${\mathrm{RV-A}}_{\text{ED}}$ and the MRI-derived RV-EDV also justifies the use of $A_{\text{ED}}$ instead of EDV [141]. A small RV end-diastolic area relative to the RV long-axis length (i.e., unaltered geometry and size) in the presence of a high ${\mathrm{VTI}}_{\text{TR}}$ (high RV systolic pressure and relatively low RA pressure) yield a high ${\mathrm{LAI}}_{\text{RV}}$ which indicates a good RV adaptability to increased afterload (i.e., capability to increase its systolic pressure without cavity dilation and also without relevant pressure rise in the RA) suggesting good myocardial contractile abilities and the potential of the RV to improve its performance after reduction of the afterload. A large area relative to long-axis length (spherical dilation) in the presence of a relatively low ${\mathrm{VTI}}_{\text{TR}}$ yields a low ${\mathrm{LAI}}_{\text{RV}}$ which indicates impaired adaptation to higher afterload (i.e., excessive RV dilation with reduced ability to increase the systolic pressure in response to high afterload, consequently also increasing TR and RA pressure), suggesting impaired myocardial contractility and low probability for RV improvement during LVAD support. ${\mathrm{LAI}}_{\text{RV}}$ values <15 indicate a low adaptability to load, possibly insufficient to prevent RV failure, even after normalization of the LVAD-promoted vascular resistance in the pulmonary circulation [11, 139]. Fig. 1 (Ref. [9, 11, 79, 88, 91]) shows a simple ECHO-based clinical algorithm for facilitation of decision-making concerning the need for an additional RV support in LVAD candidates.

## 5. Usefulness of ECHO during VAD Implantation

TEE is useful for intraoperative exclusion of a PFO that may have been masked by increased LA pressure, as well as final evaluation of cardiac valve and RV function. Intraoperative TEE also helps in guiding cannula placement and optimization of ventricular support by the VAD.

### 5.1 Positioning of the Cannulas and Assessment of Blood Flow

Examination of inflow and outflow cannula (IC and OC, respectively) position and anastomoses is particularly important [57, 58, 142, 143, 144, 145, 146, 147]. The IC, should be parallel to the septum, directed toward the MV and not too close to the ventricular walls [58, 142, 143, 144]. Excessive IC deviation towards the septum necessitates intra-operative revision. Short-term reduction of LV volume by increasing the pump rate facilitates the confirmation of the correct position of the IC [145]. Real time 3D-ECHO allows better visualization of the IC within the LV [58].

Flow-Doppler techniques allow the assessment of blood flow at the cannulas. The normal color flow-Doppler pattern and velocities, as well as the flow alterations in pathological circumstances are shown in Table 3 (Ref. [57, 58, 139, 144, 145, 146, 148, 149, 150, 151, 152, 153, 154]).

**Table 3.** **Major echocardiographic signs for optimal and impaired left ventricular mechanical support**.

Optimal LV support [58, 145, 149]	Underfilling/Excessive unloading [139, 144, 146, 151, 152, 153]	Overfilling/Impaired LVAD support [145, 146, 150, 154]
● For CF-LVADs, the normal flow pattern at the cannulas revealed by flow-Doppler techniques is characterized by a laminar and unidirectional blood flow with systolic augmentation and peak velocity of 1–2 m/s (depending on intrinsic LV function and preload). Axial-flow shows at the IC a peak filling velocity between 0.7–2 m/s. At the OC, the normal flow peak velocity varies between 0.5–2 m/s.	● Rapid RV geometry alteration with TR increase and worsening of RV function associated with left-ward septum-shift, no AV opening, and extreme LV diameter decrease (± suction events) shortly after LVAD initiation, indicate overpumping-induced excessive LV decompression.	● The combination of low pump flow with increased power values resulting in impaired LV support can arise by LVAD malfunction or by increased afterload.
		● The major causes of LV overfilling are cannula regurgitation, cannula obstruction and LVAD malfunction.
● For pulsatile pumps, the peak flows in the IC and OC are usually higher than for CF-LVADs, but not beyond 2.3 m/s.	● The combination of low pump flow with normal or low power (and low PI) values indicates reduced LVAD preload (i.e., under-fillling) which is most commonly related to RV failure, significant TR, or hypovolemia.	● LVAD malfunction can result from primary failure of different device components or by thrombi inside the pump and/or the cannulas.
● A neutral septum position between the ventricles denotes adequate LV decompression.		
High right-sided heart pressure, dyssynchrony from inter-ventricular contraction delay, ischemic wall motion abnormalities, post-sternotomy state, pericardial constriction, and RV pacing can complicate the assessment of septum position.	● Underfilling of the supported LV also shrinks the cavity size and can cause obstruction of the IC orifice by suction of the myocardial or chordal structures.	● In CF-LVADs cannula regurgitation can occur in patients with high systemic vascular resistance. In pulsatile VADs, failure of the one-way valves, lead to regurgitation.
● Reduced aortic valve opening frequency and degree.	● ECHO signs of underfilling are:	● Cannula obstruction can result from mal-position, kinking or thrombosis (complete, partial, or intermittent).
	- reduction of LVEDD	
	- reduction of LA size	● Aliased high velocity turbulent flows at the cannulas orifices (i.e., >2.3 m/s peak inflow and >2.1 m/s peak outflow for pulsatile LVADs and >2 m/s peak inflow and outflow for CF-LVADs) indicate narrowing. Low diastolic flow velocity and high systolic/diastolic flow velocity ratio suggest thrombosis-related LVAD malfunction.
	- leftward septum shift	
	- reduction of AV openings	
	- signs for reduced filling pressure	
	(decrease in E-wave velocity, decrease in E/E’ ratio prolongation of E-DT)	
		● Major ECHO signs for impaired LVAD support are LV dilation, more frequent AV openings, rightward septum-shift, increased spontaneous “ECHO-contrast” in the left-sided heart, LA dilation, new appearance or increase of MR, RV and RA diameter increase, increasing TR with increase in the peak velocity of the TR-jet, as well as inferior vena cava dilation.
		● Differentiation of systemic vasoconstriction from mechanical problems is crucial because of the completely different therapeutic approaches. The AV response, which will stay closed in patients with severe vasoconstriction, is particularly useful for differentiation.

LVAD, left ventricular assist device; CF, continuous flow; LV and RV, left and right ventricle, respectively; IC and OC, inflow and outflow cannula, respectively; TR, tricuspid regurgitation; AV, aortic valve; ECHO, echocardiography; LVEDD, left ventricular end-diastolic diameter; PI, pulsatility index; LA and RA, left and right atrium, respectively; E-wave, early diastolic peak mitral flow velocity; E/E’, early diastolic peak mitral flow to mitral annular velocity ratio; E-DT, E-wave deceleration time; MR, mitral regurgitation.

### 5.2 Optimization of LV Support and Monitoring of the Right-Sided Heart 

During LVAD implantation, TEE is mandatory for intraoperative assessment and optimization of LV unloading, monitoring of RV size, geometry and function, as well as close observation of possible changes in TR and/or RA size [139].

The goal of LVAD implantation is to increase cardiac output while decreasing filling pressures. The main parameters for assessment and monitoring of LV unloading are ventricular and atrial septum position, LVEDD and AV opening [144, 146]. After LVAD initiation, sudden excessive LV unloading can induce immediate alterations of RV geometry associated with RV dysfunction (up to acute RVF). RV overdistension should therefore be absolutely prevented. Neutral ventricular and atrial septum position is essential for prevention of RV and/or TV ring geometry alterations [146, 148, 149]. Preoperative RV pathological remodeling and dysfunction is often reversible by LV pressure relief-induced reduction of RV afterload plus enhanced pulmonary vasodilation medical therapy [53, 155]. If LVAD initiation remains without any impact on RV size, geometry and function, additional insertion of a temporary RVAD might be useful because its placement concomitantly with LVAD implantation increases the postoperative survival chances compared to delayed RVAD implantation [156, 157, 158]. LVAD-induced decrease of RV afterload is usually followed by regression of TR. Nevertheless, persistence of more than moderate TR has a negative impact on RV improvement and facilitates further aggravation of TR, which often will need a subsequent surgical correction [148, 149, 156]. Simultaneous LVAD insertion and TVr in patients with pre-operative >moderate TR associated with RVF can facilitate RV reverse remodeling and spare patients from long-term BiVAD [159, 160].

After starting the LVAD, TEE is mandatory for re-evaluation of AV coaptation, because the reduction of LV pressure increases the transvalvular ΔP, which can aggravate a pre-operatively often underestimated AR [139, 161]. This became particularly important with the increasing use of CF devices where recirculation through the incompetent AV is more severe than in pulsatile pumps [162]. Regurgitation can be triggered and/or progressively aggravated if the OC is attached too close to the AV because this can induce distortion of the valve and, by increasing the local pressure, will also increase the diastolic transvalvular ΔP and also facilitate aortic root dilation [148, 161]. By impeding the LVAD support, AR hinders the reversal of congestive HF and therefore, patients with AR ≥grade 2, especially those with thin leaflets, require AV repair, closure or bioprosthesis at the time of LVAD insertion [56, 148].

LVAD initiation can create a pressure gradient from the RA to the LA, with right-to-left shunting in the presence of a PFO. Intraoperative TEE is therefore mandatory because PFO needs to be closed during VAD surgery [148, 163]. To improve the visualization of that shunting by TEE it is useful to induce a short-term shifting of the atrial septum to the left by increasing the LVAD flow [148]. After starting the LVAD, TEE is also necessary for guiding pump speed adjustment for optimizing LV unloading without any septum shift and, if possible, with maintaining a systolic AV opening at least once every 3 heart cycles [57, 163, 164]. Even if a maximum attainable LVAD support could be more advantageous for promoting LV reverse remodeling and increasing patient exercise capacity, sub-maximal support in order to keep a near physiologic AV function should be preferred in outpatients, because a full support enhances the risk of AV leaflet fusion, suction alarms, aortic rood thrombosis and also the emergence and/or aggravation of AR by increasing the ΔP over the closed AV [164].

## 6. Surveillance of VAD Recipients by Echocardiography

ECHO is the major cardiac imaging technique for routine monitoring of VAD therapy. It is necessary for evaluation and optimization of LV support, surveillance of the right-sided heart, and early identification of certain abnormalities which could affect the results of VAD therapy. It is also a cornerstone for assessment and valuation of unloading-promoted myocardial recovery and decision-making about a possible weaning of certain patients from the VAD.

### 6.1 Control and Optimization of LVAD Support

Accurate LV support is decisive for patient outcome after LVAD implantation and ECHO is the preferred method for routine surveillance of LVAD recipients. Of major importance for evaluation of LVAD function are the septum position (particularly the interatrial septum), the status of AV opening, the flow pulsatility in the OC of axial-LVADs, TR velocity, estimation of RA pressure, as well as LVAD system output (most useful by direct TTE-derived measurement) and total cardiac output estimation [144].

Impairment of LVAD support causes LV overfilling, whereas, underfilling is most commonly related to RVF, significant TR, or hypovolemia [144, 145, 146, 150, 151, 152, 153]. The major ECHO-signs for overfilling and underfilling are described in Table 3. LV unloading is best reflected by LVEDD, septal position and AV opening which are therefore also the most often used parameters for optimization of LVAD settings [165, 166]. For CF-LVADs, the adjustment of the rotor speed should strive to bring the septum in a neutral position and to ensure that the AV opens minimally for a very short time and/or to a very low frequency at the lowest possible LVEDD [145, 165].

Regular follow-up controls of AV opening are essential for surveillance and if necessary also for readjustment of LVAD support. AV opening depends on the ΔP between the LV and the aorta. Therefore, the frequency and degree and of AV openings will increase during LVAD malfunction associated with elevated LV pressures or in case of LV functional improvement, whereas AV openings will become less frequent and smaller or will cease completely with further decline of LV contractility or during overpumping-induced pressure relief of the LV concomitantly with pressure rise inside the aorta. In case of increased afterload, the status of AV opening depends on the site of disturbance. If OC obstruction is the cause for afterload increase, the simultaneous LV systolic pressure increase and aortic pressure decrease can result in AV opening every cycle [144]. If the increased afterload is caused by increased resistance in the systemic circulation, the aortic and LV pressure will increase concomitantly, and the AV may remain closed [144]. Persistently high pump flow in the setting of a low systemic cardiac output state raises concern for de novo severe AI [146].

### 6.2 Search for Adverse Circumstances which can Impair LVAD Therapy

Many potentially life-threatening anomalies inside and outside de supported heart, especially tamponade, intracardiac thrombi, vegetations, AR and cannula obstruction are easily identifiable with ECHO.

Timely identification of partial, intermittent or complete cannula obstructions, resulting from kinking, malposition, thrombosis, or vegetations is decisive for achieving an optimal outcome for LVAD recipients. Excessive unloading-induced reduction of LV size which increases the risk of IC obstruction by cardiac structures (“suckdown” of papillary muscles, trabeculations, septum and chordal structures) plus preload reduction by tamponade or dehydration, which also increase the susceptibility for IC orifice obstruction, are easily demonstrable by ECHO [57, 145]. In some patients with unloading-promoted amelioration of LV myocardial contractility, the increasing amount of blood ejected by the LV into the aorta can lead to excessive unloading which necessitates readjustment of the LVAD function to avoid suction events. In patients with CF-LVADs, low diastolic flow velocity and high systolic/diastolic flow velocity ratio suggest thrombosis-related LVAD malfunction [154]. If a misalignment between flow and ultrasound beam direction or a relevant SV provided by the supported LV can be excluded, intermittent low peak flow velocity may suggest LVAD malfunction. Low velocities at the IC may indicate thrombosis inside the IC. Backflow at the IC indicates malfunction of the device. Whereas in pulsatile devices, the malfunction of the one-way valve leads to regurgitation, in CF-LVADs regurgitation occurs when pumps cannot overcome the systemic vascular resistance. For the IC the most optimal Doppler-angle is provided by apical views but measurements can be impaired by reverberation-artifacts from the pump. For the OC in the ascending aorta, the best visualization by TTE is often provided by right parasternal views but adequate imaging is usually quite challenging [148, 151].

Continuous ECHO controls are important for the monitoring of AV function because relevant AR may occur also months after LVAD insertion and newly developed AS, as a result of cusp fusion, can also be a complication of LVAD support [145, 167, 168]. Because the lack of AV opening is an important risk factor for thrombosis, ECHO-surveillance of LVAD recipients with irrelevant AV opening should be particularly focused on the search for LVOT, AV and aortic root thrombi [168, 169].

In LVAD recipients, AR can be present also during LV systole if LV pressure remains below the aortic pressure. Conventional severity grading of AR using the vena contracta proximal isovelocity surface area and the jet-width/LVOT-diameter ratio could underestimate the severity of AR because it does not consider the pancyclic nature of AR-jets in LVAD recipients [167]. Newly developed ECHO parameters like systolic-to-diastolic peak velocity ratio (S/D-ratio) and OC diastolic acceleration and can be more reliable for AR grading in LVAD-supported patients [167].

The evaluation of LVEDD, AV opening-time and mitral inflow E-DT changes during ECHO ramp-tests (changes of device speed) can also be helpful in diagnosing CF-LVAD malfunction in the setting of pump thrombosis [170]. Nevertheless, small thrombi can induce false negative results, whereas high mean arterial pressure or AR can generate false positive ramp-tests [171].

### 6.3 Surveillance of Right-Sided Heart during LVAD Support

Even though LVAD initiation can lower the PVR already intraoperatively, and a durable LVAD support can further diminish the PVR and thereby also promote reverse remodeling and functional improvement of the RV, neither early nor late RVF after LVAD insertion are completely evitable, even by an additional use of enhanced pulmonary vasodilative therapy [139, 172]. Continuous monitoring of the right-sided heart is therefore mandatory and TTE is particularly useful for early detection of RV anatomical and functional alterations [9, 53, 54]. Progressive increase of RVEDD, RA volume and TR are key indicators for imminent or already present right-sided HF and additional signs for non-optimal LV unloading suggest that the alteration of RV function could be the consequence of inadequate LV mechanical support [9, 53, 54]. Persistence of right-sided heart dilation and no reduction of TR, despite optimal LV unloading, indicate either reduced RV myocardial contractility or insufficient reduction of RV hemodynamic overload or both. Main causes for RV overload despite proper LVAD function are pressure overload by high pre-capillary vascular resistance (irreducible by LV unloading) and/or volume overload (more often caused by severe kidney dysfunction and/or TR). In such patients, normal or reduced PVR indicate that reduced contractile function must be the major cause for RV dysfunction. Reduced ${\mathrm{LAI}}_{\text{RV}}$ values in these patients can additionally confirm the existence of an impaired RV contractile function [9, 54].

Compared to the prediction of early RVF, the prediction of late RVF is more challenging, particularly in patients supported by a CF-LVAD in the presence of a CRT-defibrillator prior to the LVAD implantation which was found associated with significantly higher incidence late RVF [173, 174]. Given that the underlying pathomechanisms of this more frequent complication is not clarified, closer long-term ECHO monitoring of the right-sided heart is of particular importance in these patients.

### 6.4 Evaluation of Cardiac Improvement during VAD Support

By lowering the myocardial wall-tension and optimization of blood flow into vital organs, LVADs and BiVADs can remove the major pathophysiological stimuli for cardiac remodeling and interrupt the vicious circle of ventricular dilation and reduction of the efficiency of myocardial contraction [89, 90, 91, 92, 93, 94]. All this facilitates reverse remodeling even in patients with chronic NICMP and can be accompanied by clinically relevant reversal of LV structural and functional alterations allowing sometimes even removal of the VAD. Weaning from long-term VADs was initially performed only in patients with acute forms of HF [95]. This is quite understandable because the reverse of acute HF during a longer VAD support is not unusual whereas, until quite recently, cardiac remodeling processes and functional alterations in chronic HF and were thought to be progressive, unidirectional and irreversible.

The first worldwide elective explantations of LVADs in patients with idiopathic DCM were carried out in Berlin, in 1995 (4 adult men, age 47 ± 8 years, pre-implant LVEDD 70 ± 1 mm, LVEF 13 ± 2%, HF duration before LVAD implantation 3.5 ± 0.9 years, LVAD support between 5 and 9 months) [3, 96, 97]. One of those patients is still asymptomatic, other two survived more than 15 years with their native heart, and 1 patient (free from HF recurrence) died due to sepsis, 2.6 years after weaning [3, 96, 97, 98]. At the end of 1999, already 23 patients with idiopathic DCM were weaned in Berlin from a pulsatile LVAD and in 2002 the Berlin group explanted their first CF-LVAD [4, 99]. In 2006 the Harefield group [5] reported similarly good LVAD explantation results in 11 patients with idiopathic DCM confirming the initially highly disputed feasibility of elective weaning from a long-term LVAD also for patients with pre-implant end-stage chronic NICMP who show relevant and stable LV reverse remodeling and functional improvement [4, 99, 100, 101]. Meanwhile, weaning from long-term VADs has proved to be an implementable clinical option with potential long-term successful results in adults and children, even in those with chronic HF before VAD implantation, and even if recovery remains incomplete [7, 94, 102, 103, 104, 105, 106, 175]. Because recovery rates were higher in pulsatile pumps it was initially presumed that pulsatile LVADs may provide more optimal unloading for recovery [107]. However, the less frequent explantations of CF-LVADs might also be related to the more restrictive weaning criteria introduced in certain centres, nearly simultaneously with the increasing use of CF-LVADs [175].

**Fig. 2.**
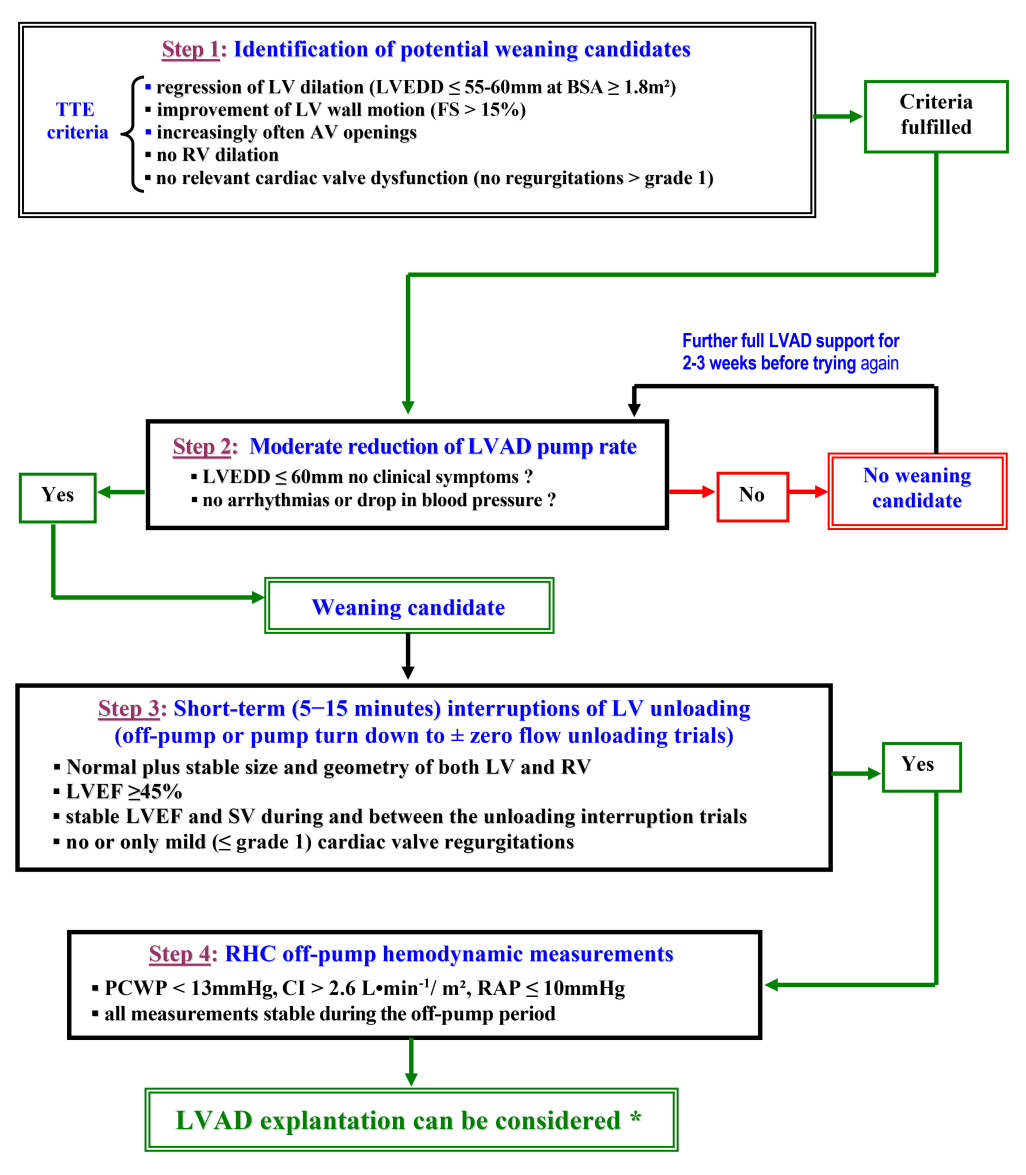
**Major steps for evaluation of cardiac improvement in LVAD recipients [6, 91, 94, 102, 107, 111, 119]**. TTE, transthoracic echocardiography; LV and RV, left and right ventricle, respectively; LVEDD, LV end- diastolic diameter; BSA, body surface area; FS, fractional shortening; AV, aortic valve; LVAD, LV assist device; LVEF, LV ejection fraction; SV, stroke volume; RHC, right heart catheterization; PCWP, pulmonary capillary wedge pressure; CI, cardiac index; RAP, right atrial pressure. * particularly in patients with adequate renal, hepatic, and pulmonary function.

**Fig. 3.**
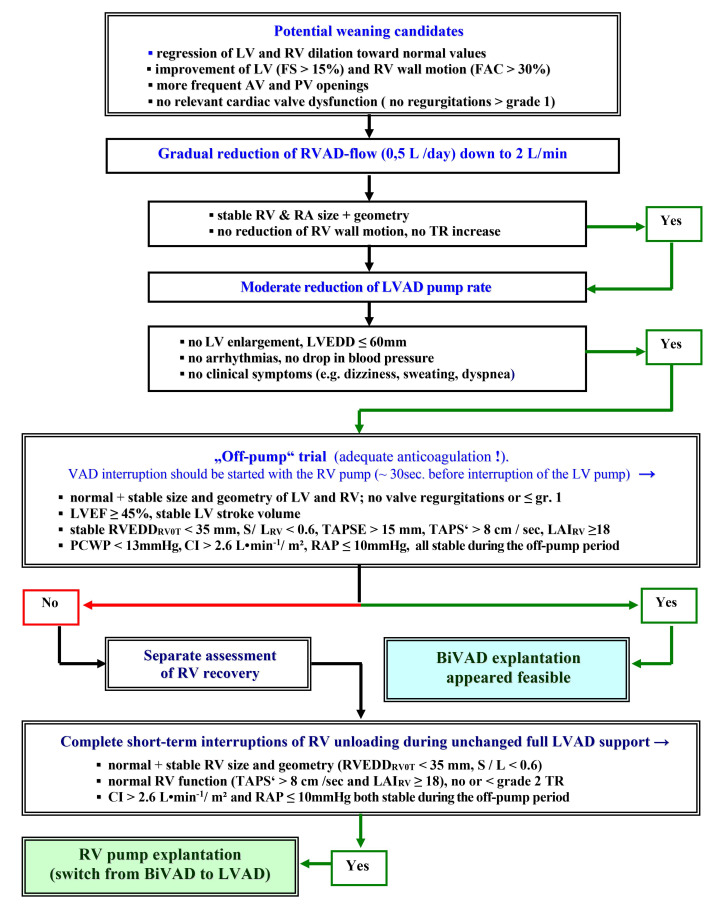
**Recovery assessment and weaning from biventricular assist devices [91, 94, 102, 107, 119]**. LV and RV, left and right ventricle, respecively; FS, fractional shortening; FAC, fractional area change; AV, aortic valve; PV, pulmonary valve; LVAD, RVAD and BiVAD, LV, RV and biventricular assist device, respectively; RA, right atrium; TR, tricuspid regurgitation; LVEDD, LV end-diastolic diameter; TAPSE and TAPS’, tricuspid annulus peak systolic excursion and velocity, respectively; ${\mathrm{RVEDD}}_{\text{RVOT}}$, end-diastolic diameter at the RV outflow tract; LAI, load adaptation index; PCWP, pulmonary capillary wedge pressure; CI, cardiac index; RAP, right arterial pressure; S/L, short/long axis ratio.

ECHO is the major tool for evaluation of myocardial recovery during LVAD support with a key role in both selection of weaning candidates and weaning decision-making [6, 54, 81, 94, 108, 109]. Regular TTE screenings with normal LVAD support are necessary for identification of potential weaning candidates. Potential weaning candidates are those with signs of LV reverse remodeling (including normalization of the LVEDD), improvement of wall motion (LV fractional shortening >15%), no relevant cardiac valve regurgitations and no RV dilation during full LVAD support [89, 90, 91]. Repeated short-term interruptions of LV unloading (off-pump or turn-down trials) are essential for recovery assessment. Whereas pulsatile LVADs allow optimal assessment of recovery during repeated short-term complete pump-stops (true off-pump trials), complete stops of CF-LVADs lead to retrograde blood flow into the LV (volume overload) which induces misleading LVEDD increase and reduction of the diastolic arterial pressure that, by reducing the LV afterload, can lead to overestimations of LV systolic function. The retrograde flow during off-pump trials is a major negative factor that can interfere with successful CF-LVAD explantation. Therefore, for such pumps, rotor-speed reduction (turn-down trials) to values resulting in close to zero flow in one cardiac cycle is better than a complete stop of the device [3, 4, 5, 57, 81, 94, 109]. Fig. 2 (Ref. [6, 91, 94, 102, 107, 111, 119]) and Fig. 3 (Ref. [91, 94, 102, 107, 119]) show the major steps for assessment of cardiac recovery in VAD-supported patients.

Before the first interruption of VAD support, a stepwise ECHO-guided pump-rate reduction is useful to determine whether complete interruptions are feasible. If a gradual reduction of the support already provokes dizziness, sweating or arrhythmias, a complete stop of VAD support is both risky and useless. If the patient stays asymptomatic but the LVEDD rises beyond 60 mm, and/or the right-sided heart shows morphological and/or functional instability (RA dilation, increasing TR, SV reduction), VAD support interruption trials are also senseless, because the patient is at present not a weaning candidate [94].

In weaning candidates, ECHO-assessment of recovery is usually based on the findings gained during repeated short-term zero-unloading trials (pulsatile pump stop or CF-pump turn-down until reaching a ±zero flow) [3, 4, 81, 109, 110, 111, 112, 113, 175]. After a prolonged nonstop mechanical assistance of the ventricle it is advisable to avoid (at least initially) any risk of myocardial exhaustion, which might impair the possibly still ongoing recovery processes. Therefore, in Berlin, from the beginning of the weaning program, all explanted long-term VAD recipients underwent preoperative recovery assessment exclusively at rest [111, 175]. Although at rest the reliability of assessment might be restricted by the lack of information about inotropic reserves and cardiac adaptation to stress, the weaning results obtained in Berlin appeared not affected by that, insofar as the results reported by groups who additionally used stress ECHO and/or exercise testing were by no means better [81, 111, 114, 175]. Nevertheless, particularly dobutamine stress ECHO (DSE) can provide useful additional information, which could be helpful in weaning decision-making [81, 114]. The critical level to define the presence of contractile reserve is defined as an increase of the absolute LVEF of more than 5% during DSE [81]. A potential disadvantage of DSE can be its possible negative impact on an ongoing myocardial recovery process. Future studies are therefore necessary to establish the real value of DSE for weaning decision-making.

Before LVAD support interruption trials, heparin administration is necessary to prevent thrombus formation inside the pump (60–100 IU/kg according to the prothrombin time) [6, 175]. Patients with heparin-induced thrombocytopenia should receive a synthetic thrombin inhibitor (e.g., argatroban infusion 2 μg/kg/min started 1 h before off-pump trials) [91]. Complete pump-stop or pump turn-down to ±zero flow should be considered carefully in patients with a history of stroke/transient ischemic attack, in those with hemolysis or difficulties in anticoagulation therapy, and are usually contraindicated when pump thrombosis is suspected (even in the absence of LVAD-related mechanical problems) [91, 94].

ECHO evaluation of cardiac reverse remodeling and functional recovery should be as extensive as possible with inclusion of tissue-Doppler imaging (TDI) and STE measurements [111, 115, 175]. Advantages of STE are its ability to differentiate between active and passive movement of different myocardial segments, the angle independency of measurements, the possibility to quantify intra-ventricular asynchrony and dyssynergy, as well as the possibility to evaluate visually not assessable components of myocardial function (particularly longitudinal deformation) [75, 114, 116, 117, 118]. A major limitation of STE is the dependence on image quality. Therefore, not all suitable parameters for weaning decisions can be reliably measured in all patients especially if Doppler and STE examinations had to be performed during rotor-speed reduction (as in case of CF-LVAD recipients), instead of complete pump stop [106].

ECHO and RHC are fundamental cornerstones for weaning decisions [81, 113, 117, 175]. The predictive value of ECHO data for the stability of post-explant heart function are shown in Table 4 (Ref. [4, 5, 6, 7, 54, 81, 91, 94, 102, 106, 111, 118, 120, 175]). At rest, and without LVAD support, LVEF 45% plus normal LVEDD are weaning requirements [81, 94, 106, 111, 119]. Their stability after maximum improvement over the next 2 to 4 weeks between, and also during follow-up off-pump tests, appeared predictive for long-term post-explant outcome in patients with a CI >2.6 L/min/$m^{2}$ and a RA pressure ≤10 mmHg measured during an pre-explant off-pump RHC examination [94, 102, 106, 111, 175]. Whereas in LVAD candidates the LVEF is less reliable for evaluation of LV dysfunction because of the misleading impact of secondary MR leading to overestimation of systolic function, LVEF becomes reliable in weaning candidates, where the absence of relevant MR is preconditioned [81, 94, 120]. Off-pump TDI- and STE-derived measurements for assessment of LV function are also helpful for weaning decisions especially if their stability after maximum improvement is also taken into consideration. In borderline cases, STE off-pump data including intraventricular synchrony and synergy, as well as rotational mechanics of contraction can be helpful for ultimate weaning decision-making [94, 116, 118]. After maximum improvement, assessment of the stability of LV size, geometry and function also during moderate LV loading by reduction of the VAD support for several hours or days can facilitate weaning decisions in hospitalized patients [94, 102]. RHC-derived normal and also stable hemodynamic parameters during the final pre-explant off-pump trial are mandatory for a decision in favour of VAD explantation, but these parameters do not allow prediction of durable cardiac stability without VAD support. However, in LVAD recipients with less than 5-year duration of HF before pump support and with normalization of RHC-derived hemodynamic parameters obtained during interruptions of the LVAD support, certain ECHO parameters collected during pre-explant off-pump trials appeared highly predictive for post-explant long-term cardiac stability even if chronic NICMP was the underlying cause for the end-stage HF [93, 116].

**Table 4.** **Role of echocardiography for pre-explant prediction of post-explant cardiac stability in weaning candidates with evidence of cardiac recovery from chronic non-ischemic end-stage heard failure after prolonged left ventricular mechanical support**.

ECHO data obtained at rest during pre-explant LV support interruption trials			
Indicative for successful weaning (≥5 years cardiac stability)*		Indicative for HF recurrence during the first 3 years after explantation*	
Individual or combined parameters	Predictive value	Individual or combined parameters	Predictive value
LVEF ≥45% at the last pre-explant ${\mathrm{off-pump}}^{†}$ trial	74%–79% [6, 94, 175]	LVEF 35%–45% at the last ${\mathrm{off-pump}}^{†}$ trial	87%–88% [6, 94]
Stable LVEF ≥45% (no reduction until explantation)	80%–86% [6]	LVEF 35%–45% in patients with >5 yrs duration of HF	Up to 100% [6]
LVEF ≥45% and normal LVEDD at the last trial	78%–88% [6, 175]	Unstable LVEF ≥45%	Up to 90% [94]
LVEF ≥45% and ${\mathrm{RWT}}_{\text{LV}}$ ≥0.38 at the last trial	87%–88% [6]	LVEF ≥45% without LVEDD normalization or persistence of with LV geometry alterations (${\mathrm{RWT}}_{\text{LV}}$ <0.38)	89% and 82%, respectively [6, 94]
Stable LVEF ≥45% and Sm ≥8 cm/s	87% [94, 111]	LVEF ≥45% but unstable geometry (${\mathrm{RWT}}_{\text{LV}}$ reduction of >8%, or ${\mathrm{S/L}}_{\text{ED}}$ increase of >10% at the last off-pump* trial)	87% and 85%, respectively [94]
Stable LVEF ≥45% plus normal and stable LVEDD	94% [94, 175]	LVEF ≥45% with reduced or unstable wall motion velocity (Sm <8 cm/s or Sm alteration of >10% during the last trial	83% and 90%, Respectively [94]
Stable LV SV (i.e., stable PW Doppler-derived VTI in the LVOT)	▪ These data are alone not predictive for long-term freedom HF recurrence [94]	LVEF 45%–50% with concurrent MR grade I–II (possible misleading overestimation of LVEF)	▪All are validated risk factors for early recurrence of HF after LVAD removal [94]
Absence or ≤grade 1 AR and/or MR	▪ Nevertheless, all of them are required pre-conditions for successful weaning [94, 111]	Systemic ${\mathrm{AP}}_{\text{d}}$ ≤50 mmHg (possible overestimation of LVEF)	▪ Currently there are no accurate figures available for their predictive value for early recurrence of HF after LVAD explantation [94]
Absence of or <grade 2 TR and/or PR		Relevant LV diastolic stiffness despite optimal LVEF (≥45%)	
No RV dilation (RVOT-EDD <35 mm and ${\mathrm{S/L}}_{\text{RV}}$ axis-ratio <0.6)		SV reduction and/or asynchrony/dyssynergy of LV contraction	
		RV size and geometry alterations and/or deficient RV adaptation to increased afterload during the last off-pump* trial	
Global radial, circumferential and longitudinal LV strain and strain rate. LV synchrony and synergy of contraction	Their usefulness is undisputed. Their PV has not yet been established [94]	TR (new appearance or accentuation) with or without increase in jet velocity (evidence of pulmonary arterial pressure increase as an answer to higher resistance in the pulmonary circulation) during the last off-pump trial	

le,respectively; HF, heart failure; LVEF, LV ejection fraction; LVEDD, LV end-diastolic diameter; Sm, peak systolic wall motion velocity (measured by the tissue Doppler at the basal LV posterior wall; SV, stroke volume; VTI, velocity-time integral; LVOT, LV outflow tract; AR, aortic valve regurgitation; MR, mitral valve regurgitation; PR, pulmonary valve regutgitation; RVOT-EDD, RV outflow tract end-diastolic diameter; ${\mathrm{S/L}}_{\text{RV}}$, RV short/long axis ratio; ${\mathrm{RWT}}_{LV,}$ end-diastolic LV relative wall thickness ([septum + LV posterior wall thickness]/LVEDD); ${\mathrm{AP}}_{\text{d}}$, diastolic arterial pressure; PV, predictive value. * Additional details related with the below items can be found in the references: [4, 5, 7, 54, 81, 91, 102, 106, 118, 120]; ^†^the term “off-pump” here stand for interruption of LV support (i.e., pump stop for pulsatile devices or rotor-speed reduction to values resulting in close to zero flow in one cardiac cycle for CF device.

## 7. Conclusions

ECHO is indispensable for assessment of cardiac anatomy and function in patients referred for HTx or VAD implantation (as BTT or DT) due to end-stage HF related to chronic NICMP. In VAD candidates, ECHO is the first-line screening tool for detection of cardiac risk factors, like PFO, thrombi, valvular abnormalities (e.g., AR and relevant TR) and endocarditis that affect the LVAD therapy, as well as identification of patients who necessitate mechanical support also for the RV.

ECHO is also required for intraoperative guiding of device implantation and optimization of its supporting function, postoperative surveillance of VAD support, surveillance of the right-sided heart in LVAD recipients, search for signs of myocardial recovery and assessment of its clinical significance, as well as for weaning decision-making in patients with relevant cardiac improvement.

Because the preoperative prediction of possible persistence or even aggravation of RV dysfunction during LVAD support is still one of the most difficult problems, a major future objective should be the further validation of the newly introduced ECHO-based composite parameters which focus on RV adaptability to increased loading conditions, and to include into established RHF risk scores, based exclusively on parameters reflecting the severity of HF-related multi-organ dysfunction, also ECHO measurements which reflect the right-sided heart anatomy and function. All this would improve the preoperative prediction of post-implant RHF in LVAD candidates.

Thanks to ECHO, which has decisively contributed to the key finding that prolonged LVAD support can trigger and further promote myocardial reverse remodeling and improvement of ventricular function up to levels which allow successful LVAD explantation, even in patients with pre-implant chronic NICMP, the previous opinion that end-stage chronic cardiomyopathy is irreversible could be refuted. In patients with normalized and stable RHC-derived hemodynamic parameters measured during short-time decommissioning of the LVAD, ECHO parameters of heart anatomy and function (including their stability between and during the LVAD support interruption trials) can predict post-explant freedom from HF recurrence.
